# Comparison of Five Serological Methods for the Detection of West Nile Virus Antibodies

**DOI:** 10.3390/v16050788

**Published:** 2024-05-15

**Authors:** Philipp Girl, Kathrin Euringer, Mircea Coroian, Andrei Daniel Mihalca, Johannes P. Borde, Gerhard Dobler

**Affiliations:** 1 Bundeswehr Institute of Microbiology, 80937 Munich, Germany; philippgirl@bundeswehr.org (P.G.); kathrineuringer@bundeswehr.org (K.E.); gerharddobler@bundeswehr.org (G.D.); 2 Central Institute of the Bundeswehr Medical Service Munich, 85748 Garching, Germany; 3 Institute for Infectious Diseases and Zoonoses, Department of Veterinary Sciences, Faculty of Veterinary Medicine, LMU Munich, 80539 Munich, Germany; 4 Division of Infectious Diseases, Department of Medicine II, University Medical Centre Freiburg, Faculty of Medicine, University of Freiburg, 79106 Freiburg im Breisgau, Germany; 5 Department of Parasitology and Parasitic Diseases, University of Agricultural Sciences and Veterinary Medicine of Cluj-Napoca, 400372 Cluj-Napoca, Romania; mircea.coroian@usamvcluj.ro (M.C.); amihalca@usamvcluj.ro (A.D.M.); 6 Praxis Prof. Borde and Kollegen, 77704 Oberkirch, Germany; 7 Department of Infectious Diseases and Tropical Medicine, LMU Center of Medicine, 80336 Munich, Germany; 8 Department of Parasitology, University of Hohenheim, 70599 Stuttgart, Germany

**Keywords:** anti-NS1-IgG WNV, WNV, mosquito-borne, flavivirus, neutralization test

## Abstract

The West Nile Virus (WNV), a member of the family *Flaviviridae*, is an emerging mosquito-borne flavivirus causing potentially severe infections in humans and animals involving the central nervous system (CNS). Due to its emerging tendency, WNV now occurs in many areas where other flaviviruses are co-occurring. Cross-reactive antibodies with flavivirus infections or vaccination (e.g., tick-borne encephalitis virus (TBEV), Usutu virus (USUV), yellow fever virus (YFV), dengue virus (DENV), Japanese encephalitis virus (JEV)) therefore remain a major challenge in diagnosing flavivirus infections. Virus neutralization tests are considered as reference tests for the detection of specific flavivirus antibodies, but are elaborate, time-consuming and need biosafety level 3 facilities. A simple and straightforward assay for the differentiation and detection of specific WNV IgG antibodies for the routine laboratory is urgently needed. In this study, we compared two commercially available enzyme-linked immunosorbent assays (anti-IgG WNV ELISA and anti-NS1-IgG WNV), a commercially available indirect immunofluorescence assay, and a newly developed in-house ELISA for the detection of WNV-NS1-IgG antibodies. All four tests were compared to an in-house NT to determine both the sensitivity and specificity of the four test systems. None of the assays could match the specificity of the NT, although the two NS1-IgG based ELISAs were very close to the specificity of the NT at 97.3% and 94.6%. The in-house WNV-NS1-IgG ELISA had the best performance regarding sensitivity and specificity. The specificities of the ELISA assays and the indirect immunofluorescence assays could not meet the necessary specificity and/or sensitivity.

## 1. Introduction

The West Nile Virus (WNV) is an emerging mosquito-borne flavivirus causing potentially severe infections in humans and animals involving the central nervous system (CNS) [1,2,3]. WNV belongs to the *Flaviviridae* family, the genus *Flavivirus*, and is a member of the Japanese encephalitis virus (JEV) sero-complex, with the St. Louis encephalitis virus (SLEV), Murray Valley encephalitis virus (MVEV), and Alfuy virus (ALFV) among others [3,4,5]. WNV was first isolated in 1937 from a woman in the West Nile District of Uganda who was suffering from a mild febrile illness, and it has subsequently been associated with sporadic cases of disease as well as large outbreaks in Africa, Eurasia, Australia, and the Middle East [6,7,8]. Today, WNV has the widest distribution of all arboviruses and is present, with different WNV subtypes, on all continents except Antarctica [7]. In addition to humans, WNV can be found in different animals including birds, horses, sheep, reptiles, cats, and rodents. [9,10,11,12,13,14,15,16,17,18,19]. The virus cycles in a natural transmission cycle between birds and mosquitoes [8,20,21]. Also, wild and domestic animals serve as sentinels. Data show a high false-positive serological reactivity in ELISA compared to the specific virus neutralization test (NT) [22,23]. Although many mosquito species have been shown to be capable of transmitting WNV, the most important vectors are mosquitoes of the genus *Culex* [8,20].

The incubation period in humans is not precisely known but probably ranges between 2 and 15 days [20,24]. The disease is asymptomatic and goes unnoticed in most cases (>80%). However, in humans and horses, a symptomatic course may occur with symptoms ranging from mild febrile illness to severe generalized infections involving the central nervous system (CNS) [12,13,20,25]. The clinical WNV diagnosis is complicated because there is no specific clinical sign of the disease in any of the affected species [3]. Diagnosis is therefore based primarily on laboratory tests such as virus detection by isolation or RT-PCR or verification of specific antibodies by serology [3]. Viral cultures and RT-PCR can be performed from different samples (serum, CSF, urine, and tissue). When samples are collected early in the course of disease, these methods can be used for confirmation of a WNV infection if positive. However, the probability of detecting WNV by molecular testing is low because many patients present in the CNS stage for the first time to a healthcare provider who then initiates diagnostics [26]. Laboratory diagnosis of a WNV infection is therefore usually made by the verification of WNV-specific IgM antibodies in serum and/or CSF [26]. IgM antibodies can usually be detected within 4 to 7 days after initial exposure and may persist for more than a year [8,27]. WNV IgG antibodies can be detected shortly after IgM antibodies and persist for many years after infection. While the presence of WNV-specific IgM in blood or CSF is usually a good indicator for a recent infection, the exclusive presence of the IgG antibody indicates a previous infection because of its long persistence [26].

Cross-reactive antibodies following infection with other flaviviruses or vaccination (e.g., tick-borne encephalitis virus (TBEV), yellow fever virus (YFV)) remain a major challenge in diagnosing flavivirus infections. Virus NTs are therefore still considered as reference tests for the detection of specific WNV antibodies and are therefore utilized to differentiate between the specific flaviviruses causing the infections [3,8,26]. Neutralization assays are elaborate, time-consuming and are therefore provided by specialized biosafety level 3 facilities. A simple and straightforward assay for the differentiation and detection of specific WNV IgG antibodies is not available but would be highly desirable and especially help in serosurveys checking for previous infections. Such an assay might improve the laboratory diagnosis of WNV infections, especially in geographic areas with circulation and transmission of multiple flaviviruses and/or high vaccination coverage against specific flaviviruses.

In this study, we compared two commercially available enzyme-linked immunosorbent assays (Anti-West Nile Virus ELISA [IgG] and Anti-West Nile Virus NS1 ELISA [IgG], both Euroimmun AG, Lübeck, Germany), a commercially available indirect immunofluorescence assay (IIFA, Flaviviren-Mosaik 1, Euroimmun AG, Lübeck, Germany), along with a newly developed in-house ELISA for the detection of WNV-NS1-IgG antibodies. All four tests were performed and analyzed side by side, and then compared to an in-house NT to determine both the sensitivity and specificity of the four test systems. We assessed their potential as an alternative to conventional NT methods.

## 2. Materials and Methods

### 2.1. Serum Samples

We used a total of 111 serum samples. Of these, 37 were classified as WNV antibody positive and 74 as WNV antibody negative. Both positive and negative results were based on neutralization tests against WNV, which is generally accepted as a gold standard for the differentiation of flavivirus antibodies. Potentially cross-reacting flavivirus infections were excluded by titration against the most common flaviviruses. All the sera used in the study could be clearly classified into one of the three groups listed below. The tested samples were grouped in the following groups:

(1) Group 1: 53 samples from anonymized blood donors in Northwestern Romania as part of a seroprevalence study by Coroian et al. were obtained. All sera were tested for antibodies to WNV, Usutu virus (USUV), and tick-borne encephalitis virus (TBEV) [28] in the respective NTs. Antibodies against yellow fever virus (vaccination) and dengue viruses (travel-associated infection) were excluded by pre-testing against these viruses by indirect immunofluorescence. WNV-suspect sera from the Coroian et al. study, that exclusively reacted positively in the WNV-NT, were classified as WNV-true positives in this comparative study.
(2) Group 2: 13 samples were provided by the German National Consultative Laboratory for TBEV from patients with an acute TBEV infection. In all the sera, TBEV-specific IgG and IgM antibodies were detected using IIFA and the infection was subsequently confirmed in a TBEV-NS1-ELISA and a TBEV-NT [29,30].
(3) Group 3: 45 serum samples were obtained from blood donors from TBEV-endemic and WNV non-endemic areas located in the federal state of Baden-Wuerttemberg in Southern Germany. To analyze the TBEV status (vaccinated, infected, naive), the samples were pre-screened using TBEV-IIFA (IgM and IgG), TBEV-NS1-ELISA, and analyzed by TBEV-NT. All samples were negative for TBEV-specific IgM-antibodies, which excluded an acute or recent TBEV infection. A total of 8 samples were negative in the IIFA (IgM and IgG) and in the NT, and therefore did not contain any TBEV-specific antibodies. Meanwhile, 37 samples were positive in the TBEV-IIFA IgG and NT. In order to distinguish between vaccination- and infection-induced antibodies, all positive sera were tested in the NS1-IgG ELISA. The 36/37 IIFA- and NT-positive sera were found to be negative. In conclusion, the antibodies had been induced by a TBEV vaccination. One serum tested positive in the NS1-ELISA, consistent with a TBEV infection prior to vaccination.

### 2.2. WNV-NT

The NT was performed as a micro-NT using a standard procedure as described previously [29,31,32]. In brief, WNV (strain 101; kindly provided by R. Shope, former Yale Arbovirus Research Unit) was cultured in Vero E6 cells. Virus stocks were prepared, titrated, and subsequently stored at −80° C until further use. Then, 96-well cell culture plates (Greiner bio-one, Frickenhausen, Germany) were used to perform the NTs. After complement inactivation at 56° C for 30 min, the serum samples were assayed in duplicates at a dilution of 1:10 and two-fold dilutions (up to 1:1280), diluted in Minimal Essential Medium (MEM, plus Non-Essential Amino Acids Solution and Antibiotic-Antimycotic Solution; all Invitrogen, ThermoFisher Scientific, Darmstadt, Germany). A defined positive and negative control along with a cell control and viral back titration were added to each 96-well plate. Thereafter, 50µL of virus dilution containing 50 TCID of WNV virus were added to each serum dilution and incubated for one hour at 37° C. Subsequently, 10^4^ cells were added and incubated for five days at 37° C (5% CO_2_). Titer determination was performed visually after discarding supernatants and fixing and staining the 96-well plates in 13% formalin/PBS, containing crystal violet (0.1%). The antibody titer corresponding to the highest serum dilution showing complete inhibition of virus growth, as determined by the absence of a cytopathic effect (CPE) in both wells, was indicated. In case there was a difference among the titers in the duplicates, the test was only accepted as valid if the titer was differentiating by only one two-fold step. The lower titer was used for analysis in this situation. In the case of more than a two-fold difference in the serum, the testing was repeated. Thus, samples were classified as either “NT-negative” (titer < 10) or “NT-positive” (titer ≥ 10).

### 2.3. Indirect Immuno-Fluorescence Assay (IIFA)

IIFA was performed using a commercially available assay (Flaviviren-Mosaik 1, Euroimmun AG, Lübeck, Germany) according to the manufacturer’s instructions. Serum samples were diluted two-fold, starting at 1:10 to 1:640 and assayed for WNV-, TBEV-, YFV- and JEV-specific antibodies. For evaluation, the tests were evaluated by two experienced persons independently using a fluorescence microscope (Leica DM 5000B, Wetzlar, Germany) and classified as “positive” (characteristic cytoplasmic fluorescence visible, no fluorescence in the control field) or “negative”, respectively (no characteristic fluorescence visible, no fluorescence in the control field/uncharacteristic fluorescence in the positive and control field) as recommended by the manufacturers. In the case of uncharacteristic fluorescence in positive and control field samples, these were classified as “negative” as well.

### 2.4. WNV-ELISA (ELISA)

WNV-ELISA (Anti-West Nile Virus ELISA [IgG], Euroimmun AG, Lübeck, Germany) was performed according to the manufacturer’s instructions. For photometric measurement, a Tecan Sunrise (Tecan, Männedorf, Switzerland) was used. The measured optical density (OD) was converted into a ratio based on the calibrator values. Samples with a ratio of less than 0.8 were considered negative for WNV-specific IgG antibodies, samples with a ratio between 0.8 and 1.1 were categorized as borderline, and samples with a ratio of 1.1 and greater were classified as positive. To ensure better comparability with the NT, borderline ELISA results were scored as positive in this study.

### 2.5. WNV-NS1-ELISA (NS1-ELISA)

WNV-NS1-ELISA (Anti-West Nile Virus NS1 ELISA [IgG], Euroimmun AG, Lübeck, Germany) was performed according to the manufacturer’s instructions. A Tecan Sunrise (Tecan, Männedorf, Switzerland) was used for the photometric analysis. This test was a “for research use only” product. Analogous to the WNV-ELISA, the measured OD was converted into a ratio depending on the calibrator values. Samples with a ratio of less than 0.8 were ranked as negative for WNV-NS1-specific IgG antibodies, samples with a ratio between 0.8 and 1.1 as borderline, and samples with a ratio of 1.1 and more as positive. Again, borderline results were considered as positive.

### 2.6. In-House WNV-NS1-ELISA (IH-NS1-ELISA)

The in-house WNV-NS1-ELISA was prepared and performed with a method analogous to an established procedure for TBEV [30]. In brief, 96-well polystyrene plates (Nunc Immuno MaxiSorp, Thermo Fisher Scientific, Waltham, MA, USA) were coated overnight with recombinant WNV NS1 antigen (The Native Antigen Company (Kidlington, UK), WNV-NS1-100) at a concentration of 0.25 μg/mL in carbonate buffer (0.6 M, pH 9.6). After the wells were blocked with gelatin (PanReak AppliChem, Darmstadt, Germany) in phosphate-buffered saline (PBS), 100 μL of each test serum (diluted 1:100) was added and incubated for 1 h at 37 °C. Plates were then washed three times and then 100 μL of horseradish peroxidase (HRP)-conjugated detection antibody (polyclonal rabbit anti-human IgG-HRP, Dako, Jena, Germany) was added to each well and incubated for 1 h at 37 °C. After another three wash cycles, 100 μL of the substrate tetramethylbenzidine (TMB; Substrate Chromogen ready-to-use, Dako, Jena, Germany) was added and the reaction was stopped after 8 min by adding 50 μL of 0.5 M sulfuric acid. Optical density was measured using a Tecan ELISA reader (Infinite F50, Tecan, Männedorf, Switzerland). Samples were measured in triplicates with one positive and one negative control on each plate. Positive and negative controls consisted of pooled serum samples with positive anti-WNV IgG antibody levels tested by NT. The evaluation as positive or negative was made with respect to the mean OD of the triplicates. Samples with an OD above 0.2 were considered positive. Samples with an OD below 0.2 were validated as negative.

### 2.7. Statistical Analysis

Correlations between NT titers and ELISAs/IIFA were evaluated using Pearson’s regression analysis (95% confidence interval). Statistical analyses were performed using GraphPad Prism 8 for Windows (GraphPad Software, San Diego, CA, USA).

## 3. Results

### 3.1. Detection of WNV-Specific Antibodies by NT

In the WNV-NT, 33.3% (37/111) of the samples tested positive (Titer ≥ 1:10) for WNV-specific antibodies, while 66.7% (74/111) showed no reactivity (titer < 1:10) (Table 1). No specific antibodies against any other flavivirus tested could be detected. So, all positive sera had a monospecific flavivirus reaction.

### 3.2. Detection of Antibodies with the IIFA

**Figure 1:**
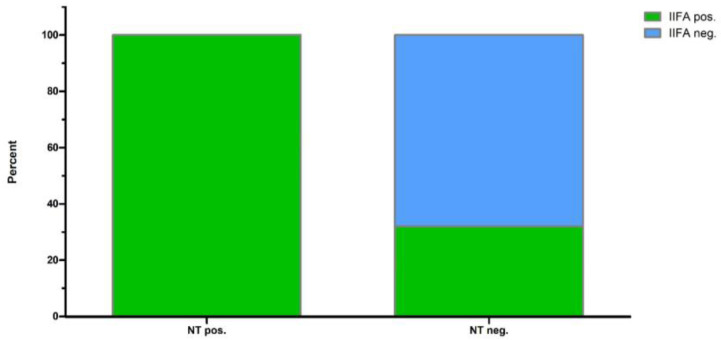
Proportionate distribution of sera determined as positive or negative in IIFA for negative and positive neutralization test (NT) results.

A total of 60 out of 111 samples tested (54.1%) were classified as positive for WNV-specific antibodies based on characteristic fluorescence in IIFA (Table 1). On the other hand, 51 samples (45.9%) were negative. Furthermore, 23 IIFA-positive samples (37.1%) tested negative in NT. All WNV NT-positive samples also showed reactivity in IIFA, corresponding to a sensitivity of 100%. On the other hand, IIFA confirmed 51 of the 74 WNV NT-negative samples, corresponding to a specificity of 68.9% (Figure 1). Some WNV-positive samples also showed weak fluorescence for one or more of the other flaviviruses tested. In contrast, none of the WNV-negative sera showed a positive reaction in IIFA for any of the other three flaviviruses tested.

### 3.3. Detection of Antibodies by ELISA

**Figure 2:**
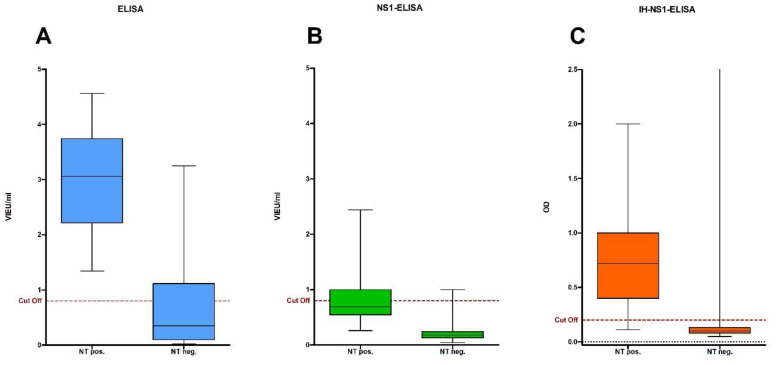
Distribution of Vienna units (VIEU)/mL determined by ELISA (**A**) and NS1 ELISA (**B**) or OD determined by IH-NS1 ELISA (**C**) for negative and positive neutralization test results (NT). The cut-off of each assay is shown as a red dashed line.

In the ELISA, 53.2% (59/111) of all tested samples showed a positive result for WNV-specific antibodies (Table 1). All NT-positive samples were correctly identified as positive, corresponding to a sensitivity of 100%. Specificity reached 70.3%, and 22 of the 74 NT-negative samples were correctly detected by the ELISA (Figure 2A).

### 3.4. Detection of Antibodies by NS1-ELISA

Only 16 of the 111 samples (14.4%) showed a positive result in the NS1-ELISA (Table 1). Compared to the NT, the NS1-ELISA achieved a sensitivity of only 37.8%, with a correct identification of 14 of the 37 NT-positive samples. At 97.3%, NS1-ELISA achieved the highest value of all four test systems in terms of specificity; only 2 of the 74 NT-negative samples were not confirmed in NS1 (Figure 2B).

### 3.5. Detection of Antibodies by IH-NS1-ELISA

Of the 111 samples tested, 39 (35.1%) showed a positive result in the IH-NS1-ELISA and 72 (64.9%) showed a negative result (Table 1). Moreover, 35 of the 37 NT-positive samples showed a positive result in IH-NS1-ELISA, correlating with a sensitivity of 94.6%. The specificity of IH-NS1-ELISA reached 94.6%, as 4 of the total 74 NT-negative samples were falsely evaluated as positive (Figure 2C).

## 4. Discussion

The reliable detection of WNV-specific antibodies is important, especially in epidemiology and surveillance of the spreading disease. However, robust discrimination of antibodies directed against other, potentially cross-reactive flaviviruses, or against corresponding vaccine-induced antibodies, is an increasing challenge because an ongoing emergence of flaviviruses has been observed worldwide [33,34,35,36]. The NT assay is still considered the most specific serological test method and is used as the gold reference standard [37]. At the same time, the NT has disadvantages that cannot be ignored; the test implementation and procedure are complex and the essential viable WNV virus can only be handled in BSL-3 facilities.

Various commercial ELISA and IIFA systems are available that can detect antibodies directed against WNV coat proteins. However, due to the increasing spread of the various flaviviruses, these systems are reaching their limits regarding specificity. Antibodies directed against the envelope proteins in particular are prone to cross-reactivity. Furthermore, these such proteins are usually included in the available vaccines [38,39,40]. A promising new approach to improve the specificity of the test systems is the search for antibodies directed against the non-structural protein NS1. This method has already proven successful in TBEV diagnostics, among others, and shows enormous advantages with regard to cross-reactions [30,41,42]. Another advantage of this approach is that NS1 protein is usually not included in the available inactivated vaccines, thus additionally enabling a distinction between vaccine and infection antibodies [30,41,42]. Currently, this distinction is only of interest for veterinary medicine, where there are already approved WNV vaccines for horses. But in the future, corresponding vaccines are also likely to enter the market in human medicine. In this study, we tested two well-established conventional test systems, an ELISA and an IIFA, as well as two NS1-based ELISA systems and evaluated their performance against the NT as the reference standard.

The conventional WNV-ELISA achieved a high sensitivity (100%) but only a moderate specificity (70.3%). In a recent study by Gómez-Vicente et al., the WNV-ELISA reached a sensitivity of 91.1% and a specificity of 100% [43]. However, the sera that were positive in both the assays being compared were also considered true positives and were not additionally verified in NT. Notably, false positive results, e.g., due to cross-reactive antibodies against other flaviviruses, cannot be detected in this way. In our study, all presumably WNV-positive samples (group 1) were additionally tested in the NT, and only NT-positive samples were classified as true positive. This could result in the difference shown in the specificity assessment between the two studies. In Gómez-Vicente et al., the WNV ELISA also indicated a slightly lower sensitivity than in our study. This could also be due to the different definition of safe-positive sera; Gómez-Vicente et al. also ranked sera from PCR-positive WNV patients as true positives. It must be kept in mind that these patients were still in an early stage of the disease, and IgG antibodies were absent or present in low titers and consequently the WNV ELISA had not detected them as positive yet.

IIFA also showed a high sensitivity of 100% and a slightly lower specificity of 68.9%. Therefore, it is considered inferior to the other test systems. As described above, there is a strong potential for cross-reactivity with antibodies to other flaviviruses. To exclude these antibodies against the flaviviruses present in Romania in the tested samples, the sera were tested in NTs against TBEV and USUV as described above. Exclusively sera with negative results against the two flaviviruses were further processed and included in the study. The other two flaviviruses, YFV and JEV, contained on the IIFA chip are not reported in Romania, and vaccination against these two viruses is uncommon in the Romanian regions and population. Thus, we conclude that infection or vaccination of the tested blood donors is unlikely, although a travel-related infection cannot be completely excluded. Therefore, the positive reactions against the other viruses on the chip are most likely false positives caused by the cross-reacting antibodies to WNV. The assumption for cross-reactivity and not for infection or vaccine-induced antibodies is supported by the finding that in none of the flavivirus-positive but WNV-negative sera, a positive result against any of the other flaviviruses tested in IIFA could be detected.

NT is generally considered the most specific serologic test, but not necessarily the most sensitive. Basically, it would be possible that the NT missed some low positive sera that were correctly identified as positive in the other methods due to insufficient sensitivity. Accordingly, the calculated specificity of the compared methods (ELISAs and IIFA) would be rated too low. However, since the NT is also the most sensitive test in this comparative setting, this possibility seems unlikely.

The WNV-NS1 ELISA showed the lowest sensitivity of all the tested methods with only 37.8%, but at the same time the highest specificity of 97.3%. Specificity could be further increased by conservatively scoring the thresholds as negative, resulting in a specificity of 100% in this comparison. At the same time, however, this would result in a further drop in sensitivity to 18.9% (Figure 2B). Why this test has such a low sensitivity remains unclear but it may be due to the high specificity gained for an ELISA. Both the in-house NS1 ELISA and comparable test systems from the other studies showed a significantly higher sensitivity with good specificity [30,41,42].

The in-house WNV-NS1 ELISA achieved the best result in terms of sensitivity (94.6%) and specificity (94.6%) when considered together. Surprisingly, the IH-NS1 ELISA showed a much better sensitivity (94.6% vs. 37.8%) compared to the commercial NS1 ELISA. Both test systems are coated with the NS1 protein of WNV, but no details are available on the exact design of the commercial test system. The IH-NS1-based assay showed an equivalent specificity to that of the conventional EI NS1-ELISA of 94.6% and 97.3%, respectively.

In this comparison, none of the systems tested could match the specificity of the NT, although the two NS1-based ELISAs were very close to the specificity of the NT at 97.3% and 94.6%. The in-house WNV-NS1 ELISA had the best performance when sensitivity and specificity were combined. The conventional ELISA also used in this study showed up to 100% cross-reactivity in a previous study by Niedrig et al., when testing sera with antibodies against DENV, TBEV and YEV [44]. In our study, we repeat this finding of a strong susceptibility of the conventional ELISA regarding cross-reacting antibodies. When the sera from group 1 were considered in isolation, the ELISA achieved a specificity of at least 82.4%, which fell significantly to 70.3% when the TBEV-positive sera were added. The specificity of the IIFA fell slightly from 70.6% to 68.9%. For the two NS1-ELISAs, the specificity even increased significantly from 88.2% (NS1-ELISA) and 82.4% (IH-NS1-ELISA) to 97.3% and 94.3%, respectively.

For the analysis of sera from regions with more than one endemic flavivirus or a high flavivirus vaccination rate, a two-step testing procedure might be useful, in which sera are prescreened in the very sensitive, easy, and inexpensive ELISA and positive samples are subsequently confirmed in a highly specific procedure such as NT. Further studies should investigate the performance of NS1 ELISAs in testing more potentially cross-reacting sera. In other studies, NS1-based assays have also demonstrated significantly better resistance to cross-reactivity while maintaining good sensitivity [30,41,42]. NS1-based systems could thus replace the use of complex NTs in suitable applications even without a two-step process.

Our study included only 111 serum samples, but the samples are all well characterized and NT verified. Combining the results of the three groups (WNV-infected people without potentially cross-reacting antibodies, acute TBE sera, blood donors with/without TBEV antibodies and without WNV antibodies), we tried to represent the real current situation in most European countries. This made it possible to compare the test performance in an ideal group without potentially cross-reacting antibodies and a realistic group in which the test systems must prove themselves in everyday life. The effects of other potentially cross-reacting antibodies from infections or vaccinations with other flaviviruses such as YFV, DENV or JEV on the test systems is a matter of future research.

## Figures and Tables

**Table 1:** All micro-neutralization test (NT)-positive sera were confirmed by IIFA and ELISA, whereas IH-NS1-ELISA correctly identified 94.6% (35/37) and NS1-ELISA correctly identified only 37.8% (14/37) as positive. Of the NT-negative samples, 97.3% (72/74) and 95.9% (71/74) were correctly identified as negative by ELISA and IH-NS1-ELISA, respectively. 68.9% (51/74) and 58.1% (43/74) were correctly identified as negative by IIFA and NS1-ELISA, respectively. The IIFA, ELISA, NS1-ELISA, and IH-NS1-ELISA results had a statistically significant correlation with the NT result (*p* < 0.0001, Fisher’s exact test).

Serum Samples *n*		*WNV IIFA*		*WNV ELISA*		*WNV NS1-ELISA*		*WNV IH-NS1-ELISA*	
		Pos	Neg	Pos	Neg	Pos	Neg	Pos	Neg
Total	111	60	51	59	52	16	95	39	72
WNV NT reactive	37	37	0	37	0	14	23	35	2
WNV NT negative	74	23	51	22	52	2	72	4	70
*Sensitivity [%]*		100.0		100.0		37.8		94.6	
*Specificity [%]*		68.9		70.3		97.3		94.6	

## Data Availability

The data presented in this study are available upon request from the corresponding author. The data are not publicly available due to privacy restrictions.
